# Using Geospatial Analysis to Determine Access Gaps Among Children with Special Healthcare Needs

**DOI:** 10.1089/heq.2017.0050

**Published:** 2018-03-01

**Authors:** Pamela DeGuzman, Paige Altrui, Aubrey L. Doede, Marcus Allen, Cornelia Deagle, Jessica Keim-Malpass

**Affiliations:** ^1^ School of Nursing, University of Virginia, Charlottesville, Virginia.; ^2^ Children and Youth with Special Health Care Needs, Virginia Department of Health, Richmond, Virginia.; ^3^ Division of Child and Family Health, Virginia Department of Health, Richmond, Virginia.; ^4^ Department of Pediatrics, School of Medicine, University of Virginia, Charlottesville, Virginia.

**Keywords:** GIS, geospatial, health policy, rural health, child development, children with special healthcare needs

## Abstract

**Purpose:** To examine geospatial gaps in identification and evaluation of children with special healthcare needs (CSHCN) within public child development centers (CDCs).

**Methods:** A descriptive geospatial design was used to visually depict service gaps, proximity, and clustering of area-level sociodemographic attributes of Virginia counties, and patient-level data within each CDC.

**Results:** Geospatial analysis shows population density of uninsured children against CDC resources. Data visualization facilitates policy advocacy based on the identification of care and screening gaps for CSHCN.

**Conclusion:** This project illustrates the collaborative potential between researchers and Health Department members to identify gaps in access to care.

## Introduction

Access to care for children with special healthcare needs (CSHCN) disproportionately affects those living in rural areas. CSHCN are defined as “those who have, or are at increased risk for, a chronic physical, developmental, behavioral, or emotional condition and who also require health and related services of a type or amount beyond that required by children generally.”^1^ As many as 23% of households have at least one child with special healthcare needs, with estimates of 11.2 million of those under the age of 18 in the United States being identified as CSHCN.^2^ Children with mental, behavioral, and developmental disorders are more prevalent in small rural areas (18.6%) than in urban dwellings (15.2%).^3^ These childhood conditions are associated with poor mental health of the parents, financial and child care difficulties, and a lack of coordinated medical care and medical homes.^4^ Rural parents of CSHCN are more likely than their urban counterparts to report difficulty with transportation to appointments and lack of service availability in their region, particularly therapy and mental healthcare counseling.^5^ Rural families, who are more likely to lack resources as well as to have limited access, may, therefore, rely on safety net services provided by public health departments. These providers need the ability to assess their reach into rural areas.

Public health systems that identify and evaluate CSHCN are critical to link vulnerable children with supportive care services, particularly in rural areas. CSHCN conditions become more common as children age (18.4% ages 12–17 years old),^2^ which may be, in part, driven by delays in diagnoses related to rural geographic barriers to access.^6^ Healthy People 2020 goals now reflect structural and socioeconomic barriers to care in rural areas by their incorporation of the physical and social environment as critical focus areas within health promotion and disease prevention for all Americans.^2^ Public health providers can now use geographic information systems (GIS) software to identify potential structural environmental-level barriers by evaluating geographic gaps in access to care and utilizing these data to reduce disparities in their health districts.^7^ Visual depiction of gaps in healthcare access can aid policymakers in understanding the depth and breadth of geographic-based health disparities.

In 2016, researchers from the University of Virginia collaborated with the Virginia Department of Health (VDH) to assess geospatial gaps in care for CSHCN who were identified as part of the VDH child development centers (CDCs). VDH funds and oversees five CDCs throughout Virginia, which are codified in state legislation to provide services to CSHCN through the Maternal and Childhood Grants (12VAC5-191-210).^8^ These centers are composed of a team of healthcare professionals, brought together in one location, to provide specialized services to CSHCN and their families, including assessment, diagnosis, consultation, referral, and coordination of services. The most common disorders seen at the VDH CDCs are attention-deficit/hyperactivity disorder, speech or developmental delays, motor or other physical disorders, and autism spectrum disorder (ASD).^9^

The purpose of this study was to demonstrate how an academic–practice partnership utilized GIS to examine and identify geospatial gaps in identification and evaluation of CSHCN being served by a state public health provider. To our knowledge, this is the first study in which GIS has been used to visually identify local and regional gaps for CSHCN.

## Materials and Methods

We used a descriptive geospatial design to identify the gaps in service. This design utilizes spatial projection of geographic characteristics to visually depict the physical proximity and clustering of individual or area-level characteristics. The study was conducted with the approval of the University of Virginia Institutional Review Board.

Deidentified patient-level data, representing all CDC children seen in 2015, were collected from the five CDCs and included county of residence and age at the time of initial evaluation. We layered individual-level data over county-level socioeconomic data to better understand the sociodemographic environment. Data were gathered from the U.S. Census American Community Survey, 2014 five-year estimates.^10^

To visually depict the counties of residence for children who were seen in a CDC, a choropleth map was created using ArcGIS (v10.3) to indicate county-level number of uninsured children. The patients within each CDC's catchment area (the geographic area that is served by each CDC) contained between 200 and 1441 children per catchment area (3219 total). Individual-level socioeconomic data were limited to age and insurance status due to a lack of standardization in collection of these data points across CDC sites. Our map showed the distribution of all children seen in a CDC in 2015 on top of base maps with county-level quintiles of population of uninsured children. Counties were shaded in the base maps according to the number of uninsured children by dividing the county-level data in quintiles and indicating more dense areas with darker shading.

## Results

**FIG. 1.**
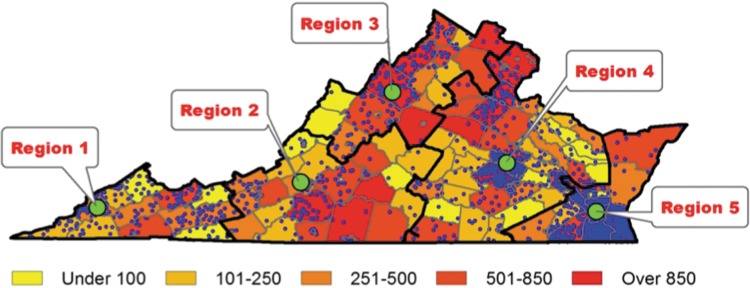
Children accessing child development centers layered over density of uninsured children <18 years old, divided by county. Green dot represents child development center. Blue dot represents one child accessing a child development center. Map created by P.C. Altrui and P.B. DeGuzman using ArcGIS v 10.3. (Sources: 2014 American Community Survey 5-Year Estimates^10^ and Department of Health Child Development Centers).

Figure 1 shows Virginia counties outlined in gray and each CDC's catchment area outlined with a heavy black border. Each small dot represents one child located within his or her residential county who accessed the CDCs in 2015. Larger dots represent the locations of each CDC. Several sections of the state show few or no patients, particularly the northeastern border of Region 5 and the central corridors along the eastern borders of Region 3 and Region 2.

Figure 1 also layers the number of children seen in a CDC over estimates of uninsured children <18 years old within each county, divided into shaded quintiles, with darker shading indicating a higher number of uninsured children. There are several counties in Regions 2 and 3 with >500 uninsured children <18 years old with few or no patients seen at their respective CDC.

## Discussion

Mapping of insurance- and location-based data demonstrates two important spatial relationships between CDCs and the populations they serve. First, it is possible to visually identify the spatial relationship between the children seen by a CDC and the locations of those CDCs. As there is only one CDC per VDH-defined region of Virginia, greater distance between CDCs and children in need of screening may reflect an access barrier. For example, the map shows a cluster of CDC patients in the northern portion of Region 4 who have been screened by a CDC, although they do not live close to that location. Although these children have been screened by a CDC despite increased travel distance, this may not be the case for all children with similar travel challenges.

Uninsured children are less likely to be diagnosed with ASD, one developmental condition screened for by CDCs.^11^ As such, our map suggests that there may be groups of children in need of CDC access who have never been screened based on location and access to care. These are areas such as the southeastern portion of Region 2 or the northeastern tip of Region 5, where there is a lack of a CDC close to that area despite the number of uninsured children.

Limitations of this study were due to the exclusive use of publicly available data sets and thus only describing the services of one provider (VDH) during a 1-year timeframe. The lack of other available data, therefore, did not allow this study to be comprehensive of every CSHCN. In addition, each map point, representative of an individual child, was only available to the county level because of a lack of address data and may neglect more subtle differences within a county. The use of data from five CDC sites, each under separate administrative structures, led to a limitation in availability of individual-level socioeconomic data, further limiting our analysis. Lack of standardized socioeconomic data collection has been acknowledged as a nationwide limitation of U.S. health disparities research.^12^

## Conclusion

By using GIS mapping software to show the state of Virginia by population density of uninsured children as well as available CDC resources, we have demonstrated the value of visualizing potential gaps in care and screening of children with developmental conditions and other special healthcare needs. This is particularly true of areas that may benefit most from additional resources, namely, those with large number of uninsured children who carry the most risk of receiving a late or no diagnosis of developmental conditions.

The map demonstrates the value of visual displays of data with the goal of making findings more relatable and understandable to the reader. Visualizing the data in this capacity makes it easier to conduct evidence-based policy advocacy and identify needs of a community or state.^13,14^ Moreover, this project illustrates the collaborative potential between researchers and Department of Health members to identify and address these gaps. To translate health disparities to policymakers, these collaborations are increasingly dependent on standardized socioeconomic data in electronic health records.^12^

In addition, this study highlights the unique attributes made available due to an academic–practice partnership. Academic researchers are able to provide technical expertise whereas public health practitioners can help frame the unique public health problems, contribute relevant translational insights, and provide the data needed for such analyses to occur. Although this study focused specifically on CSHCN within Virginia, this type of collaboration and use of GIS may be applied to other regions and health issues using similar methods as outlined here.

## Abbreviations Used

ASD: autism spectrum disorder
CDCs: child development centers
CSHCN: children with special healthcare needs
GIS: geographic information systems
VDH: Virginia Department of Health
